# Thermal transport investigation in AA7072 and AA7075 aluminum alloys nanomaterials based radiative nanofluids by considering the multiple physical flow conditions

**DOI:** 10.1038/s41598-021-87900-w

**Published:** 2021-05-10

**Authors:** Adnan, Sheikh Irfan Ullah Khan, Umar Khan, Naveed Ahmed, Syed Tauseef Mohyud-Din, Ilyas Khan, Kottakkaran Sooppy Nisar

**Affiliations:** 1 Department of Mathematics, Mohi-Ud-Din Islamic University, Nerian Sharif, 12080 AJ&K Pakistan; 2 Department of Mathematics, COMSATS University Islamabad, Abbottabad Campus, Abbottabad, Pakistan; 3 Department of Mathematics and Statistics, Hazara University, Mansehra, 21120 Pakistan; 4 Department of Mathematics Faculty of Sciences, HITEC University, Taxila Cantt, 47070 Pakistan; 5 University of Multan, Multan, Pakistan; 6 Department of Mathematics, College of Science Al-Zulfi, Majmaah University, Al-Majmaah, 11952 Saudi Arabia; 7 Department of Mathematics, College of Arts and Sciences, Prince Sattam Bin Abdulaziz University, Wadi Aldawaser, 11991 Saudi Arabia

**Keywords:** Mathematics and computing, Applied mathematics

## Abstract

Now a day’s variety of nanomaterials is available, among these Aluminum Alloys AA7072 and AA705 are significant due to their thermal, physical and mechanical characteristics. These extensively used in manufacturing of spacecraft, aircraft parts and building testing. Keeping in view the significance of nanoliquids, the analysis of methanol suspended by AA7072 and AA7075 alloys under the multiple physical flow conditions is reported. The model is successfully treated by coupling of RK and shooting algorithm and examined the results for the flow regimes by altering the ingrained physical parameters. Then physical interpretation of the results discussed comprehensively. To validate the analysis, a comparison between the presented and existing is reported under certain assumptions on the flow parameters. It is found that the results are reliable inline with existing once.

## Introduction

Thermal transport properties in different colloidal suspensions over a permeable plate is significant and due to their variety of applications in engineering, nuclear reactors, bearings lubrication and cooling, electronics and in many other industrial zone^1^. The two physical properties of a permeable plate known as suction and injection significantly affect the heat transport characteristics and shear stresses. In 2010, Ishak reported self-similar solutions for the thermal transport over a plate under the impacts of suction/injection and convective flow condition. He pointed that the self-similar solutions for steady and streamlined boundary layer flow over a permeable plate exist under the assumption that the permeable parameter and convective heat transport from the hot liquid alters with proportion of x-1/2 in which x denotes the distance from the leading edge of the surface^2^.

The study of thermal transport in hybrid colloidal suspension over a surface with uniform suction/injection by incorporating the impacts of magnetic field in the governing hybrid model is significant. They perceived that the heat transport upsurges for more magnetic colloidal suspension while decreasing behavior was reported by increasing the volumetric fraction ϕ_2_. On the basis of presented analysis, they detected that the hybrid colloidal suspension (Cu-Al_2_O_3_/H_2_O) is reliable for better thermal transport characteristics^3^. The flow of nanofluid over a chemically heated plate with suction characteristics by incorporating the Maxwell and Brinkmann correlations for thermal conductivity and density of the nanoliquid were described. Moreover, for novelty of the analysis, they retained the phenomena of viscous dissipation, radiative heat flux and chemical reaction in the constitutive model. They pointed that the heat transport rate drops for higher fraction factor and stronger Lorentz forces^4^.

Thermal and mass transportation in the nanoliquid by plugging the influences of absorption over a magnetized and radiative plate were examined by Prasad et al.^5^. They pointed the heat transfer characteristics in two different nanomaterials (Cu and TiO_2_) suspended in water. Further, the nanoliquids velocity, thermal transport and shear stresses prevailed for Cu-H_2_O in the analysis. They reported that prevailing effects of Cu-H_2_O were due to high thermal conductance of Cu nanomaterial. The heat transfer study by incorporating the effects of porosity is significant. Keeping in view the porosity effects, Maleki et al.^6^ reported the analysis of nanoliquid over a porous radiative plate. They captured the flow and heat transfer behavior in four different nanoliquids depending upon four nanomaterials (Cu, Al_2_O_3_, SWCNTs and MWCNTs) via graphical representation. The study of streamlined boundary layer flow by considering the suction/injection property captured by Sadri and Babaelahi^7^.

The heat generation or absorption analysis in the flow regimes significantly alters the heat transport characteristics. Recently, Upreti et al.^8^ pointed the heat transfer in silver based nanoliquid under the influence of heat generation/absorption with permeable effects over a porous surface. Furthermore, they examined the behavior of the nanoliquid velocity and temperature by altering the magnetic and Eckert parameters. In 2020, Shah et al.^9^ reported the effects of Hall current, viscous and ohmic dissipation in the nanoliquid squeezed inside porous sheet.

The nanomaterials are significant due to high thermal and mechanical characteristics. These materials are significantly altering the properties of the nanoliquids composed by the respective nanomaterial. Among the nanomaterials, there is a material know as Aluminum alloys in which the role of aluminum is predominant. There are two major categories of aluminum alloys which further categorized as heat treatable and non-heat treatable alloys. Aluminum alloys are extensively used in the manufacturing of spacecraft, aircraft parts, building and testing. Due to better heat transport characteristics of AA7072 and AA7075 aluminum alloys, researchers analyzed various flow models composed by aluminum alloys and found fascinating behavior of the thermal transport. The heat transfer inspection in nanoliquids composed by AA7072 and AA7075 aluminum alloys by considering the effects of variable Lorentz forces reported Sandeep and Animasaun^10^. They detected that nanoliquid composed by AA7075 alloys is better for heat transport in comparison with AA7072 composed nanoliquid.

The analysis of thermal transport in magnetized AA7075 alloys by considering the electric field strength was perceived Kandasamy^11^. Three-dimensional heat transfer characteristic in the hybrid colloidal model AA7072-AA7075/Methanol with velocity condition examined were determined by Tlili et al.^12^. They treated the model numerically and discussed the results for the flow regimes. Further significant results for nanofluids examined in^13–16^. The impacts of aluminum nanomaterial with the addition of imposed magnetic field, heat transport due to Buoyancy effects over a paraboloid of revolution, 3D unsteady radiative flow characteristics of hybrid nanofluid and the investigation of heat transfer in CuO-GO/methanol was reported in the scientific literature^17–20^.

From the critical review the literature, it is perceived that the heat transport analysis in methanol suspended by AA7072 and AA7075 aluminum alloys is not reported over a permeable plate. For novelty of the analysis, radiative heat flux, phenomena of heat generation/absorption, suction/injection, thermal and velocity slip effects are ingrained in the governing colloidal model. Then, a numerical scheme based on shooting algorithm is adopted for mathematical treatment. Then results for the velocity, thermal transport and shear stresses for AA7072-Methanol and AA7075-Methanol are presented for suction/injection and heat generation/absorption separately. Finally, outcomes of the analysis concluded in the end.

## Formulation and AA7072 and AA7075 effective models

The colloidal flow of AA7072-Methanol and AA7075-Methanol is taken over a permeable radiative plate in the presence of heat generation and absorption physical situation. The temperature at the surface is T_w_ while apart from the surface is ${T}_{\infty }$. The effects of slip condition are also considered. The velocity for suction/injection of the nanofluids is v, w while ${U}_{\infty }$ is the velocity at free stream and volume rate of heat generation/absorption expressed by the following formula in which ${Q}_{o}$ denotes the coefficient of heat generation/absorption:

$$ Q = \left\{ {\begin{array}{*{20}l} { - Q_{0} \left( {\tilde{T}_{\infty } - \tilde{T}} \right);} & {\tilde{T}_{\infty } \le \tilde{T}} \\ {0;} & {\tilde{T} < 0} \\ \end{array} } \right. $$

**Figure 1:**
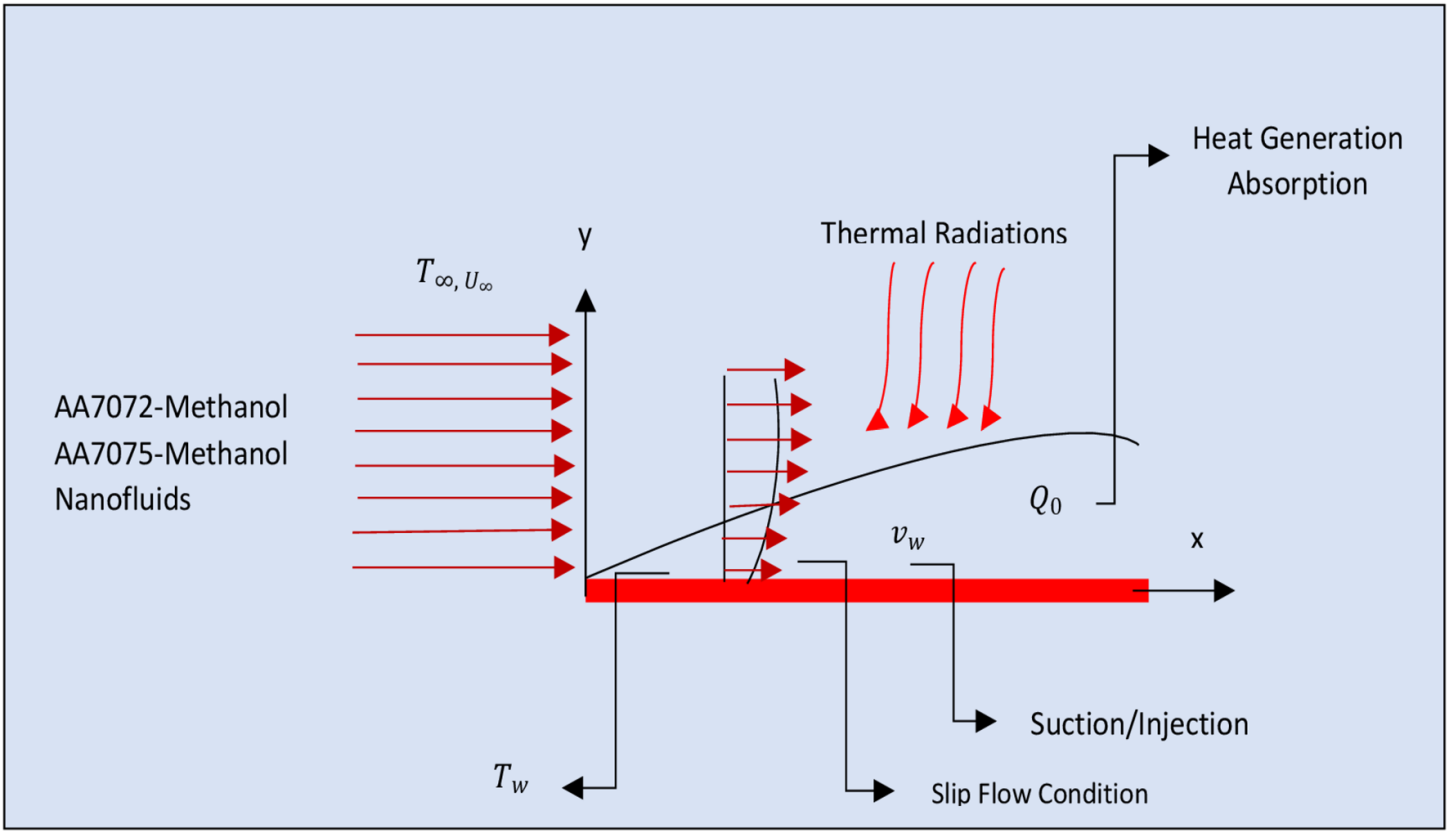
The flow of AA7072-Methanol and AA7075-Methanol over permeable plate.

Further, it is assumed that the nanomaterials AA7072, AA705 and host liquid are thermally compatible and there is no slip between them. The flow configuration of AA7072-Methanol and AA7075-Methanol is painted in Fig. 1.

Keeping in view the assumptions and various physical situations painted in the flow configuration, the governing colloidal model by plugging the influences of slip condition, permeability, radiative heat flux and heat generation/absorption described as^6^:

1 $$ \frac{{\partial \tilde{u}}}{\partial x} + \frac{{\partial \tilde{v}}}{\partial y} = 0, $$

2 $$ \tilde{u} \frac{{\partial \tilde{u}}}{\partial x} + \tilde{v} \frac{{\partial \tilde{u}}}{\partial y} - \frac{{\tilde{\mu }_{nf} }}{{\tilde{\rho }_{nf} }} \frac{{\partial^{2} \tilde{u}}}{{\partial y^{2} }} = 0, $$

3 $$ \tilde{u} \frac{{\partial \tilde{T}}}{\partial x} + \tilde{v} \frac{{\partial \tilde{T}}}{\partial y} - \left( {\tilde{\alpha }_{nf} \frac{{\partial^{2} \tilde{T}}}{{\partial y^{2} }} + \frac{{\mu_{nf} }}{{\left( {\rho c_{p} } \right)_{nf} }}\left( {\frac{{\partial \tilde{u}}}{\partial y}} \right)^{2} + \frac{{Q_{0} \left( {\tilde{T} - \tilde{T}_{\infty } } \right)}}{{\left( {\rho c_{p} } \right)_{nf} }} - \frac{1}{{\left( {\rho c_{p} } \right)_{nf} }} \left( {\frac{{ - 4\sigma^{*} T_{\infty }^{3} }}{{3k^{*} }}\frac{{\partial^{2} \tilde{T}}}{\partial y}} \right)} \right) = 0, $$

The associated flow conditions at the permeable surface and apart from it are described in the following way:

4 $$ \left. {\begin{array}{*{20}c} {\tilde{u} = \lambda_{1} \frac{{\partial \tilde{u}}}{\partial y}} \\ {\tilde{v} = - v_{w} } \\ {\tilde{T} = \overset{\lower0.5em\hbox{$\smash{\scriptscriptstyle\smile}$}}{T}_{w} + \beta_{1}^{*} \frac{{\partial \tilde{T}}}{\partial y}} \\ \end{array} } \right\}{\text{at the permeable surface}} y = 0, $$

5 $$ \left. {\begin{array}{*{20}l} {\tilde{u} \to U_{\infty } } \hfill \\ {\tilde{T} \to \tilde{T}_{\infty } } \hfill \\ \end{array} } \right\}{\text{apart from the suraface }}y \to \infty = 0, $$

where $\stackrel{\sim }{u}$ and $\stackrel{\sim }{v}$ are the velocities in horizontal and normal directions, respectively. The velocity slip, thermal slip and suction/injection are described by ${\lambda }_{1}$, ${\beta }_{1}^{*}$ and ${v}_{w}$, respectively.

The associated invertible transformations for under consideration colloidal model are described as:

6 $$ \left. {\begin{array}{*{20}c} {\eta = \left( {\frac{{U_{\infty } }}{{x\nu_{f} }}} \right)^{0.5} \tilde{y}} \\ {\beta \left( \eta \right) = \frac{{\left( {\tilde{T} - \tilde{T}_{\infty } } \right)}}{{\left( {\tilde{T}_{w} - \tilde{T}_{\infty } } \right)}}} \\ {\psi = \left( {x\nu_{f} U_{\infty } } \right)^{0.5} F\left( \eta \right)} \\ {\tilde{u} = \frac{\partial \psi }{{\partial y}}} \\ {\tilde{v} = - \frac{\partial \psi }{{\partial x}}} \\ \end{array} } \right\}, $$

where $\beta , F, \psi$ and $\eta$ described the self-similar temperature, velocity, stream function and similarity variable, respectively.

To improve the heat transfer efficiency of AA7072-Methanol and AA7075-Methanol, following effective models are adopted:

7 $$ \left. {\begin{array}{*{20}c} {\tilde{\alpha }_{nf} = \frac{{\tilde{k}_{nf} }}{{\left( {\rho c_{p} } \right)_{nf} }} = \left( {(x_{1} - 2\phi x_{2} } \right)/x_{1} + \phi x_{2} )/\left( {\left( {1 - \phi } \right)\left( {\rho c_{p} } \right)_{f} + \left( {\rho c_{p} } \right)_{s} \phi } \right)} \\ {x_{1} = k_{s} + 2k_{f} } \\ {x_{2} = k_{f} - k_{s} } \\ \end{array} } \right\}, $$

8 $$ {\mu }_{nf} = \frac {\mu_{f}} {\left( {1 - \phi } \right)^{2.5}}$$

9 $$ \tilde{\rho }_{nf} = \left( {1 - \phi } \right)\rho_{f} + \phi \rho_{s} , $$

10 $$ \left( {\rho c_{p} } \right)_{nf} = \left( {1 - \phi } \right)\left( {\rho c_{p} } \right)_{f} + \left( {\rho c_{p} } \right)_{s} \phi , $$

Thermophysical properties for alloys AA7072, AA7075 and host liquid methanol described in Table 1^12^:

**Table 1:** Liquid phase and nanomaterials thermophysical characteristics.

Thermophysical Characteristics	Unit	Liquid Phase	Nanomaterials	
		Methanol	AA7072	AA7075
*ρ*	kg/m^3^	792	2810	2720
*c*_*p*_	JK/gK	2545	960	893
*k*	W/mK	0.2035	173	222
*σ*	S/m	0.5 × 10^−6^	26.77 × 10^6^	34.83 × 10^6^

By implementing the described invertible transformations and nanofluids characteristics in the governing colloidal model, the following version of the model along with flow conditions is attained:

11 $$ \frac{{F^{\prime\prime\prime}}}{{\left( {\left( {1 - \phi } \right) + \frac{{\phi \rho_{s} }}{{\rho_{f} }}} \right)\left( {1 - \phi^{2.5} } \right)}} + 0.5FF^{\prime\prime} = 0, $$

12 $$ \frac{{((x_{1} - 2\phi x_{2} )/x_{1} + \phi x_{2} )}}{{\left( {1 - \phi } \right) + \frac{{\left( {\rho c_{p} } \right)_{s} \phi }}{{\left( {\rho c_{p} } \right)_{f} }}}}\left( {1 + \frac{4}{{3Rd\left( {(x_{1} - 2\phi x_{2} } \right)/x_{1} + \phi x_{2} )}}} \right)\beta^{\prime \prime } + 0.5PrF\beta^{\prime} + \frac{{PrEcF^{\prime \prime 2} }}{{\left( {1 - \phi } \right)^{2.5} \left( {\left( {1 - \phi } \right) + \frac{{\left( {\rho c_{p} } \right)_{s} \phi }}{{\left( {\rho c_{p} } \right)_{f} }}} \right)}} + \frac{\gamma Pr\beta }{{\left( {1 - \phi } \right) + \frac{{\left( {\rho c_{p} } \right)_{s} \phi }}{{\left( {\rho c_{p} } \right)_{f} }}}} = 0 $$

13 $$ \left. {\begin{array}{*{20}c} {F^{\prime} = \lambda F^{\prime\prime}} \\ {F = f_{w} } \\ {\beta = 1 + \beta^{*} \beta ^{\prime}} \\ \end{array} } \right\} {\text{at the}} \eta = 0, $$

14 $$ \left. {\begin{array}{*{20}c} {F^{\prime} \to 1} \\ {\beta \to 0} \\ \end{array} } \right\} {\text{at}} \eta \to \infty , $$

where heat generation/absorption corresponds to $\gamma > 0$ and $\gamma < 0$, respectively; suction/injection is $f_{w} > 0$ and $f_{w} < 0$, respectively; velocity slip parameter $\left( \lambda \right)$, thermal slip $\left( {\beta^{*} } \right)$ and $Ec$ is an Eckert number. These parameters are described by the following mathematical relations:

15 $$ \left. \begin{gathered} f_{w} = 2\left( {\frac{x}{{U_{\infty } \nu_{f} }}} \right)^{0.5} v_{w} ;\quad {\text{suction}}/{\text{injection}} \hfill \\ \gamma = \frac{{xQ_{0} }}{{\left( {\rho c_{p} } \right)_{f} U_{\infty } }};\quad {\text{heat generation}}/{\text{absorption}} \hfill \\ \lambda = \lambda_{1} \left( {\frac{{U_{\infty }^{3} }}{{x\nu_{f} }}} \right)^{0.5} ;\quad {\text{velocity slip parameter}} \hfill \\ \beta^{*} = \beta_{1} \left( {\frac{{U_{\infty }^{{}} }}{{x\nu_{f} }}} \right)^{0.5} ;\quad {\text{thermal slip parameter}} \hfill \\ Rd = \frac{{k_{f} k^{*} }}{{4\sigma T_{\infty }^{3} }}{ };\quad {\text{thermal radiation parameter}} \hfill \\ Ec = \frac{{U_{\infty }^{2} }}{{\left( {c_{p} } \right)_{f} \left( {\tilde{T}_{w} - \tilde{T}_{\infty } } \right)}};\quad {\text{eckert number}} \hfill \\ Pr = \frac{{\nu_{f} }}{{\tilde{\alpha }_{f} }};\quad {\text{Prandtl number}} \hfill \\ \end{gathered} \right\} $$

For similarity solution of the system of ODEs governing the colloidal model, parameters embedded in Eq. (15) are independent of x. These conditions fulfilled^6^ when $Q_{0} \propto x^{ - 1} , v_{w} \propto x^{{ - \frac{1}{2}}} ,\lambda_{1} \propto x^{\frac{1}{2}} , \beta_{1} = x^{\frac{1}{2}}$. Therefore, $Q_{0} = A_{1} x^{ - 1} , v_{w} = A_{2} x^{{ - \frac{1}{2}}} ,\lambda_{1} = A_{3} x^{\frac{1}{2}} , \beta_{1} = A_{4} x^{\frac{1}{2}}$. Where $A_{i}$ for $i = 1,2,3,4$ are constants. By plugging these values in Eq. (15), we arrived with the following version of the parameters:

16 $$ \left. \begin{gathered} f_{w} = 2A_{2} \left( {\frac{1}{{U_{\infty } \nu_{f} }}} \right)^{0.5} v_{w} ;\quad {\text{suction}}/{\text{injection}} \hfill \\ \gamma = \frac{{A_{1} }}{{\left( {\rho c_{p} } \right)_{f} U_{\infty } }};\quad {\text{heat generation}}/{\text{absorption}} \hfill \\ \lambda = A_{3} \left( {\frac{{U_{\infty }^{3} }}{{\nu_{f} }}} \right)^{0.5} ;\quad {\text{velocity slip parameter}} \hfill \\ \beta^{*} = A_{4} \left( {\frac{{U_{\infty }^{{}} }}{{\nu_{f} }}} \right)^{0.5} ;\quad {\text{thermal slip parameter}} \hfill \\ Rd = \frac{{k_{f} k^{*} }}{{4\sigma T_{\infty }^{3} }}{ };\quad {\text{thermal radiation parameter}} \hfill \\ Ec = \frac{{U_{\infty }^{2} }}{{\left( {c_{p} } \right)_{f} \left( {\tilde{T}_{w} - \tilde{T}_{\infty } } \right)}};\quad {\text{eckert number}} \hfill \\ Pr = \frac{{\nu_{f} }}{{\tilde{\alpha }_{f} }};\quad {\text{prandtl number}} \hfill \\ \end{gathered} \right\} $$

Therefore, when the parameters defined as in Eq. (16) then the Eqs. (11)–(14) possess similarity solutions.

The local shear stresses and heat transfer rate expressed by the following formulas:

17 $$ C_{Fx} = \frac{{\tilde{\tau }_{w} }}{{\rho_{f} U_{\infty }^{2} }}; \tilde{\tau }_{w} = \tilde{\mu }_{nf} \left( {\frac{{\partial \tilde{u}}}{\partial y}} \right) {\text{at}} y = 0, $$

18 $$ Nu_{x} = \frac{{q_{w} x}}{{k_{f} \left( {\tilde{T}_{w} - \tilde{T}_{\infty } } \right)}}; q_{w} = - \left( {\tilde{k}_{nf} + \frac{{16\sigma T_{\infty }^{3} }}{{3k^{*} }}} \right)\left( {\frac{{\partial \tilde{T}}}{\partial y}} \right) {\text{at}} y = 0, $$

After simplification following version is attained:

19 $$ Re_{x}^{0.5} C_{Fx} = \frac{{\tilde{\mu }_{nf} F^{\prime\prime}\left( 0 \right)}}{{\mu_{f} }} $$

20 $$ Re_{x}^{ - 0.5} Nu_{x} = - \left( {\frac{{\tilde{k}_{nf} }}{{k_{f} }} + \frac{4}{3Rd}} \right)\beta ^{\prime}\left( 0 \right) $$

where local Reynolds number expressed by the following mathematical relation:

$$Re_{x} = \frac{{U_{\infty } x}}{{\nu_{f} }}$$.

## Mathematical analysis of the model

The colloidal models AA7072-Methanol and AA7075-Methanol are nonlinear and tedious system of ODEs associated with multiple conditions based on imposed physical situations. So, it is tough or not even possible to solved the models for closed form solutions. Therefore, the models treated numerically. For said purpose, coupling of RK with shooting technique^21,22^ is implemented. In ordered to apply the algorithm on under consideration colloidal models, first the transformations made for the velocity and temperature equations:

21 $$ \left. {\begin{array}{*{20}c} {\tilde{b}_{1}^{*} = F, \tilde{b}_{2}^{*} = F^{\prime}, \tilde{b}_{3}^{*} = F^{\prime\prime}} \\ {\tilde{b}_{4}^{*} = \beta , \tilde{b}_{5}^{*} = \beta ^{\prime}} \\ \end{array} } \right\} $$

After plugging these transformations in model given in Eqs. (11) and (12) we attained the first order colloidal system. Then proposed algorithm is implemented and tackled the model successfully and plotted the results for the flow regimes under multiple physical imposed conditions.

## Results and discussion

### The velocity against slip parameter and suction/injection

**Figure 2:**
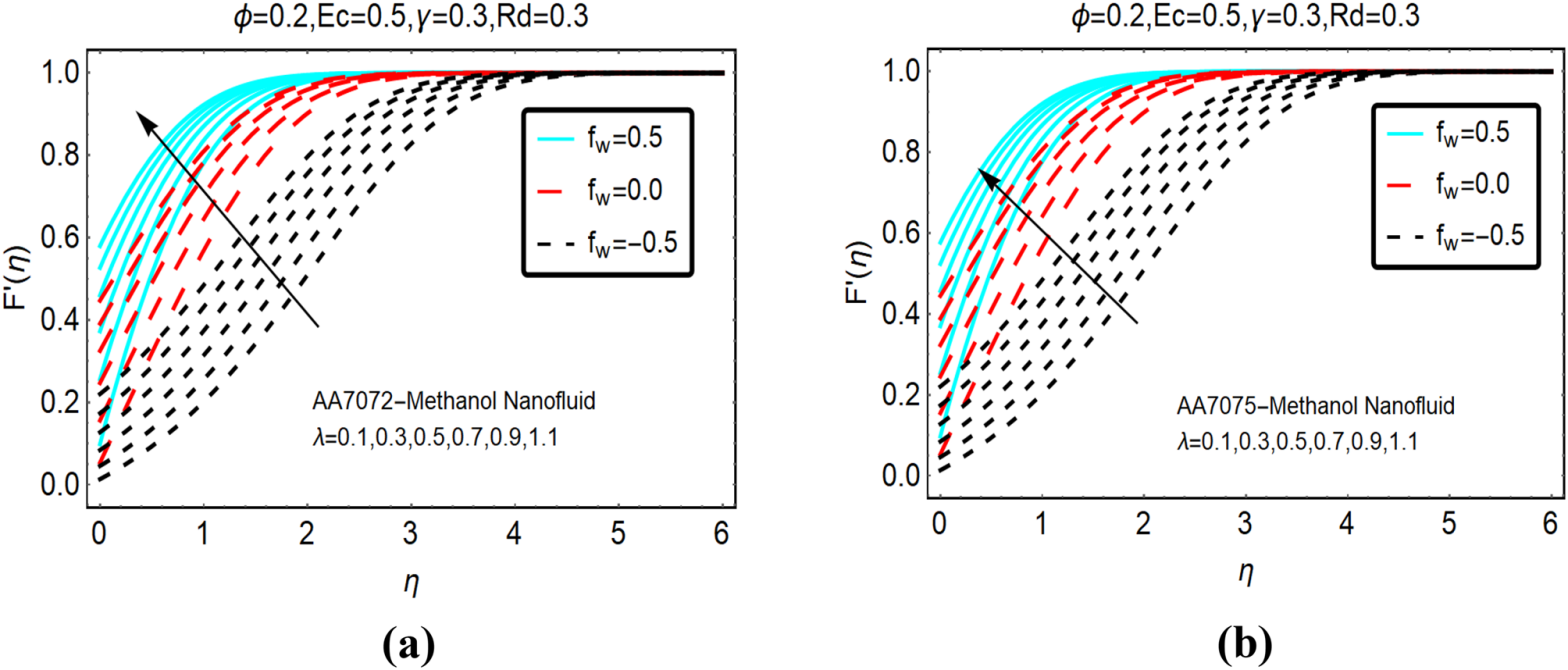
The effects of velocity slip on $F^{\prime}\left( \eta \right)$ (**a**) AA7072-Methanol (**b**) AA7075-Methanol.

The movement of AA702-Methanol and AA7075-Methanol against the velocity slip effects is decorated in Fig. 2. It worthy to mention that the fluid suction, injection and no suction/injection represented by $f_{w} > 0, f_{w} < 0$ and $f_{w} = 0$, respectively. It is pointed that the frictional force between the fluid particles and the surface reduces due to velocity slip parameter as a result abrupt increment in the fluid movement is perceived. Due to stronger slip effects in the locality of the surface, the velocity of both nanofluids rises quickly and apart from the surface these effects became minimal. Physically, injection of the fluid from the surface exerts pressure on the fluid particles near the surface. Therefore, the fluid adjacent to the surface moves very abruptly. Moreover, it is perceived that boundary layer region increases for suction case; while, declines in the boundary layer is examined for $f_{w} = 0$ and injection, respectively.

**Figure 3:**
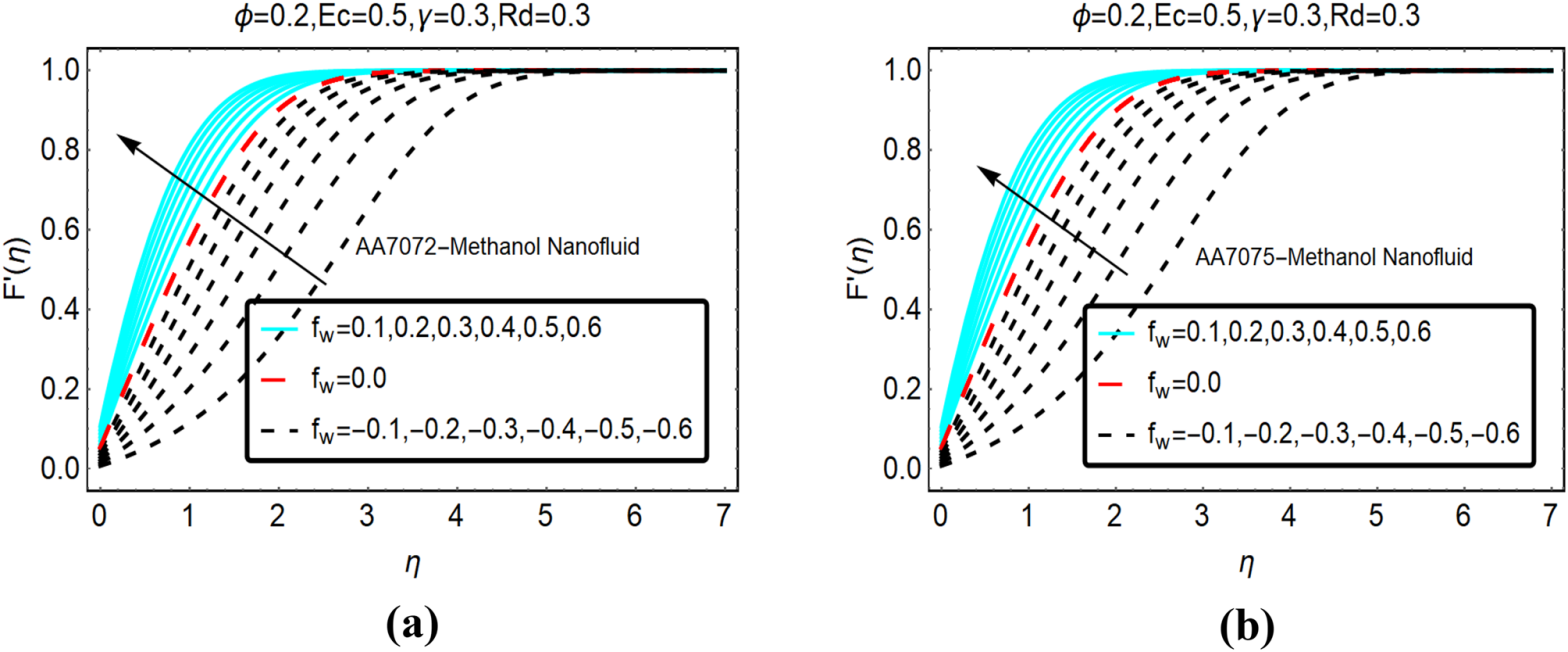
The effects of *f*_*w*_ on $F{^{\prime}}\left( \eta \right)$ (**a**) AA7072-Methanol (**b**) AA7075-Methanol.

Figure 3 portrays the fluid movement against variable suction and injection of the fluid. It is noted that for more injection of the fluid, kinetic energy of the fluid molecules increases significantly which allows the particles move abruptly. Physically, more fluid particles drag at the surface due to suction of the fluid. Due to more compactness between the fluid particles, the velocity increases quite slowly comparative to injection of the fluid.

### The temperature against Rd and thermal slip

**Figure 4:**
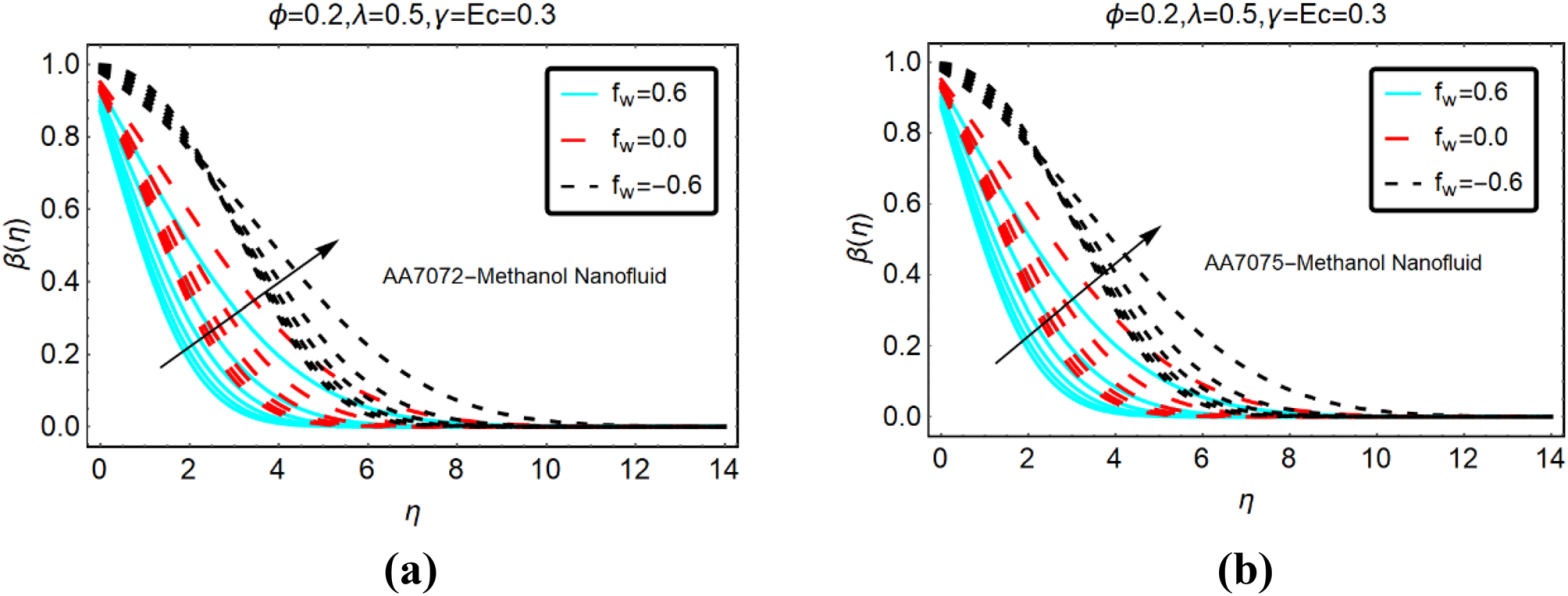
The effects of *Rd* on $\beta \left( \eta \right)$ (**a**) AA7072-Methanol (**b**) AA7075-Methanol for heat generation.

**Figure 5:**
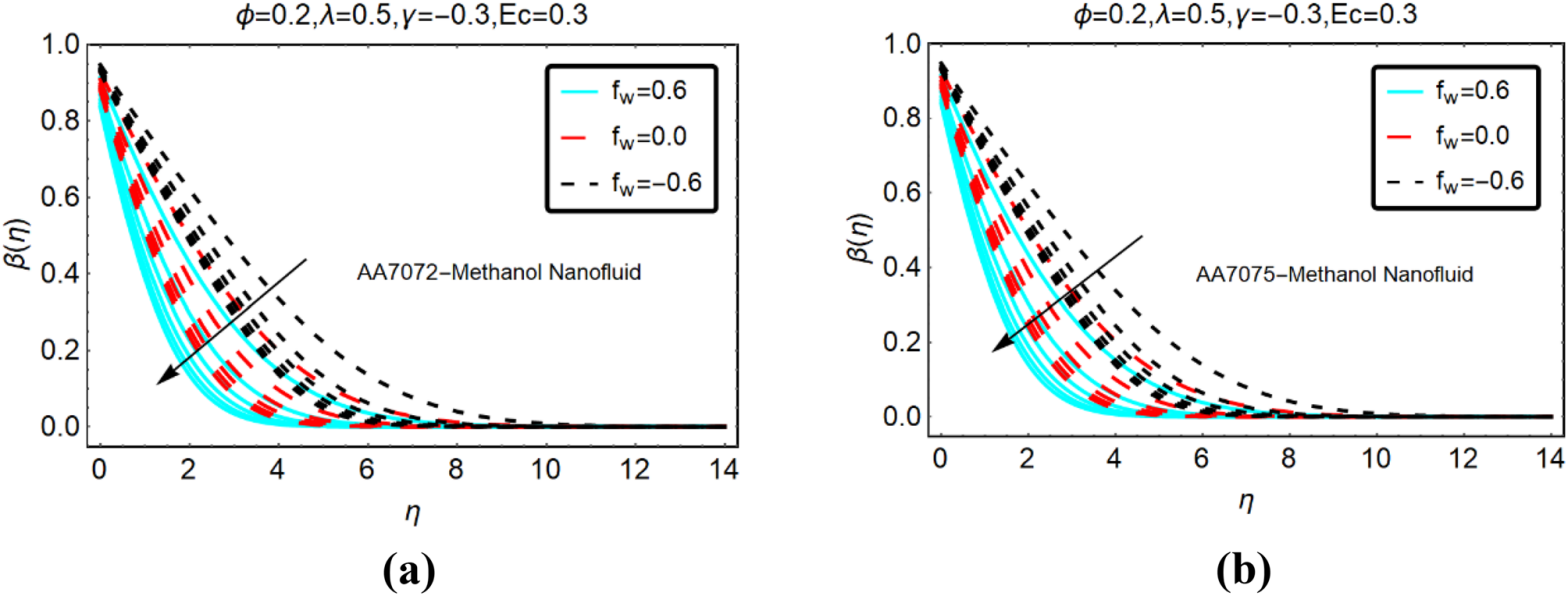
The effects of *Rd* on $\beta \left( \eta \right)$ (**a**) AA7072-Methanol (**b**) AA7075-Methanol for heat absorption.

The alterations in the temperature $\beta \left( \eta \right)$ of both nanofluids against stronger thermal radiations and heat generation and absorption are analyzed in Figs. 4 and Fig. 5, respectively. The heat generation is corresponding to $\gamma > 0$, whereas, $\gamma < 0$ stands for absorption. From the analysis, it is detected that the temperature behaviour of AA7072-Methanol and AA7075-Methanol rapidly increases due to stronger thermal radiations for heat generation. Physically, kinetic energy between the fluid particles rises against thermal radiations and due to heat generation. This allows the increase in the temperature of the nanofluids. Moreover, it is inspected that the temperature vanishes asymptotically far from the radiative surface.

The temperature alterations in radiative AA7072-Methanol and AA7075-Methanol nanoliquids under the influence of heat absorption are depicted in Fig. 5. From this, it is summarized that the temperature of the fluid declines due to heat absorption for suction case. Physically, the fluid particles become more compact due to the suction from the surface and more particles transfer at the surface. As a result, the collision between the particles reduces which leads to decrement in the fluid temperature.

**Figure 6:**
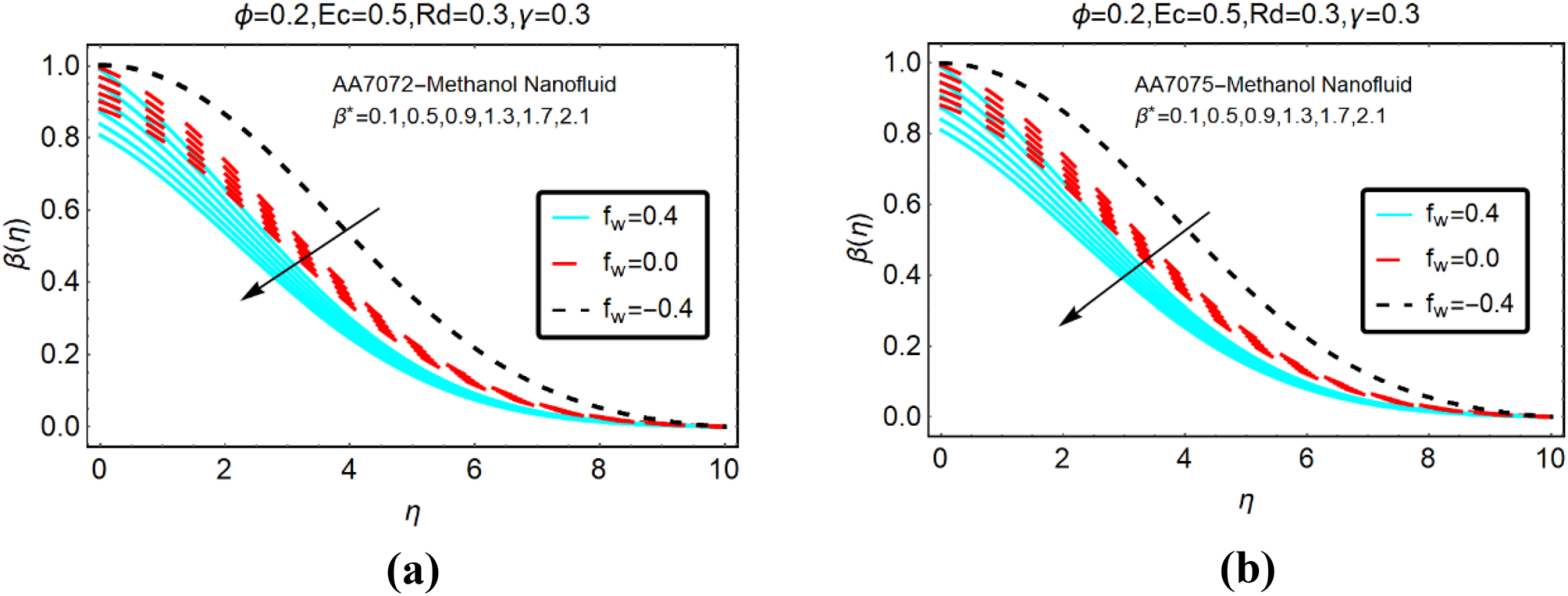
The effects of $\beta^{*}$ on $\beta \left( \eta \right)$ (**a**) AA7072-Methanol (**b**) AA7075-Methanol for heat generation.

**Figure 7:**
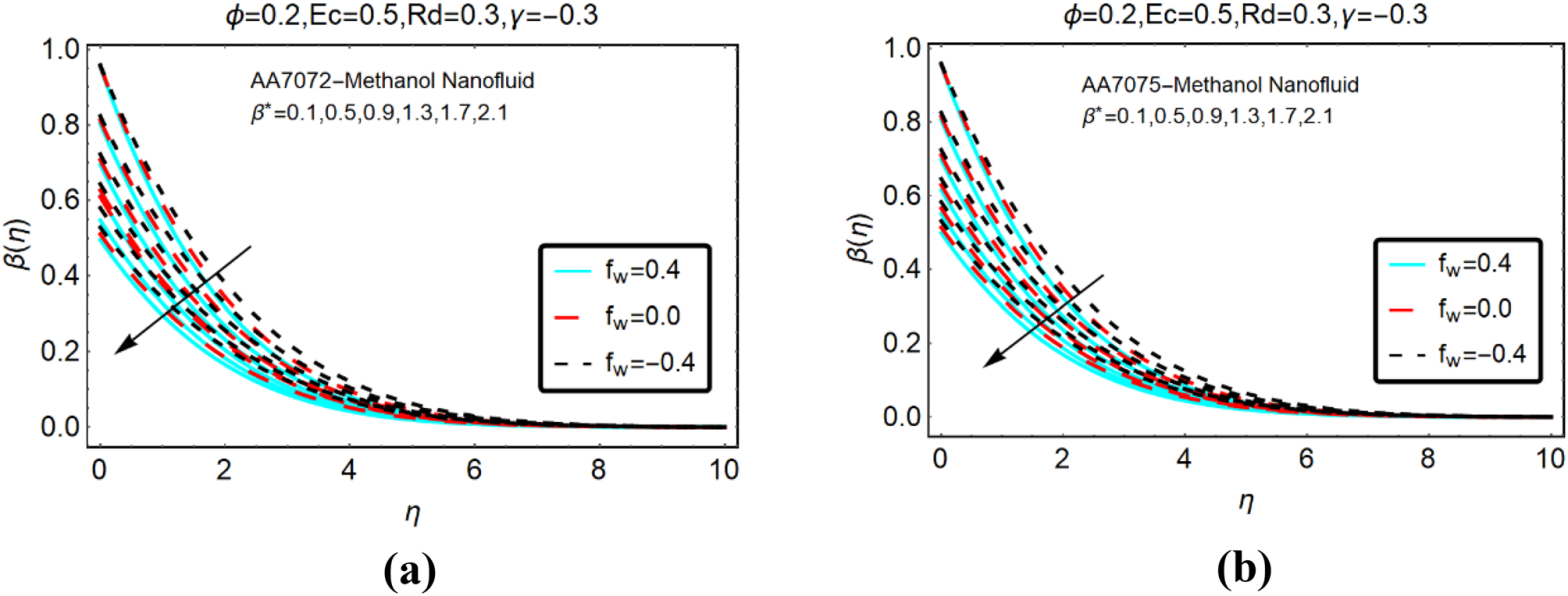
The effects of $\beta^{*}$ on $\beta \left( \eta \right)$ (**a**) AA7072-Methanol (**b**) AA7075-Methanol for heat absorption.

Figures 6 and Fig. 7 presenting the influences of slip parameter for the cases of heat generation and absorption, respectively. It is detected that $\beta \left( \eta \right)$ drops abruptly and quite slow decreasing behaviour is inspected far from the radiative surface. Physically, suction phenomena reduce the collision of the particles; consequently, the temperature drops rapidly. Figure 7 captured the temperature of AA7072-Methanol and AA7075-Methanol against thermal slip and heat absorption. The decreasing trends of the temperature $\beta \left( \eta \right)$ are investigated against stronger thermal slip effects. Physically, due to heat absorption more energy from the fluid particles is transfer at the radiative surface; ultimately the temperature $\beta \left( \eta \right)$ declines abruptly.

### The temperature against heat generation/absorption and velocity slip

**Figure 8:**
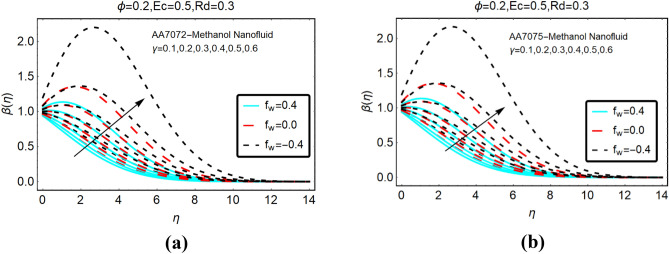
The effects of heat generation on (**a**) AA7072-Methanol (**b**) AA7075-Methanol.

**Figure 9:**
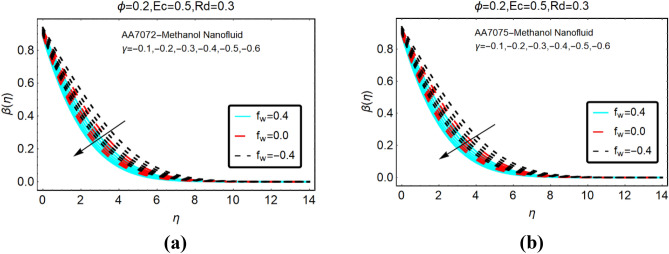
The effects of heat absorption on (**a**) AA7072-Methanol (**b**) AA7075-Methanol.

This subsection deals with the influences of heat generation/absorption and velocity slip parameters on the temperature $\beta \left( \eta \right)$ AA7072-Methanol and AA7075-Methanol. These alterations in $\beta \left( \eta \right)$ are decorated in Fig. 8. The significant increasing behavior of the temperature against stronger heat generation parameter are inspected. Physically, due to heat generation, heat energy inside the fluid rises and the surface provided extra energy to the fluid particles; consequently, the temperature rises abruptly. Further, for more injecting fluid for the surface, the energy of surroundings particles rises which lead to significant changes in the temperature. Moreover, it is detected that heat absorption reduces the temperature $\beta \left( \eta \right)$ of both nanoliquids. These results are painted in Fig. 9.

**Figure 10:**
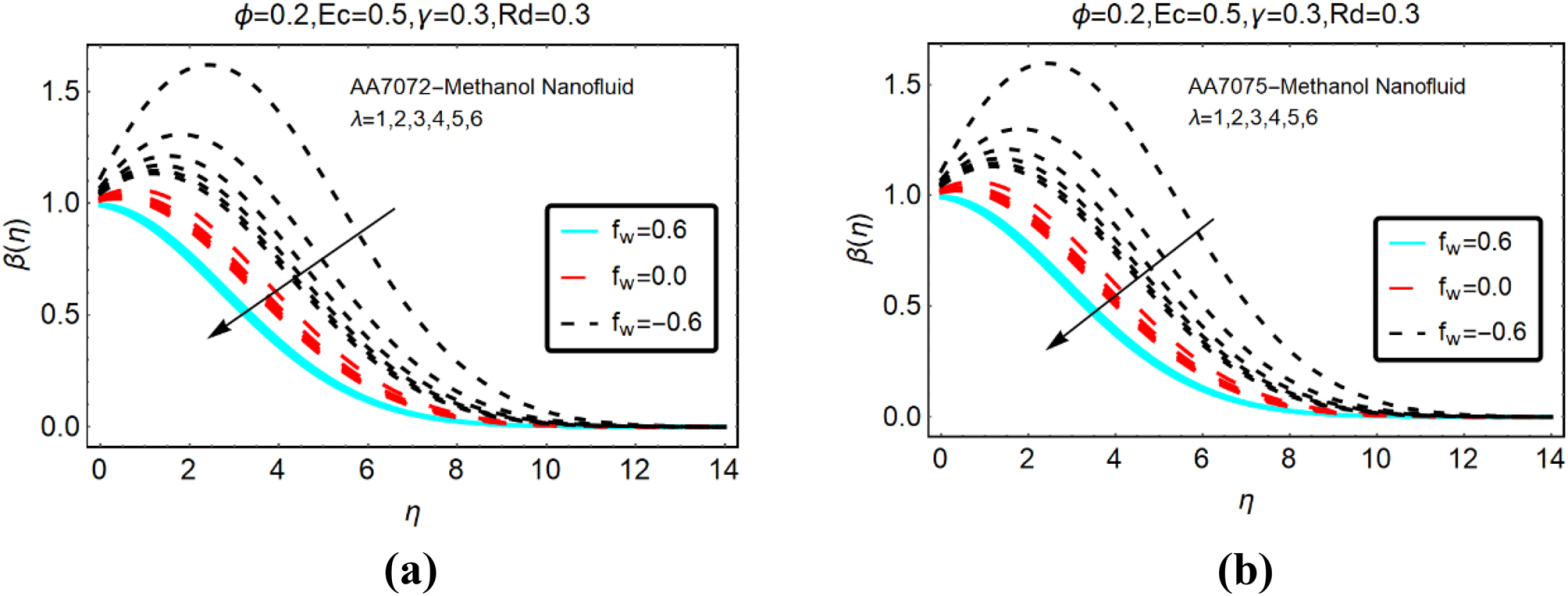
The effects of velocity slip on (**a**) AA7072-Methanol (**b**) AA7075-Methanol.

The temperature $\beta \left( \eta \right)$ of AA7072-Methanol and AA7075-Methanol against the velocity slip parameter is depicted in Fig. 10. From this, maximum decrement in the temperature $\beta \left( \eta \right)$ is noted against the injecting fluid. The thermal boundary layer thickness reduces for suction case. On the other side, decrement in $\beta \left( \eta \right)$ prevailed for injecting case.

### Shear stresses and Nusselt number

**Figure 11:**
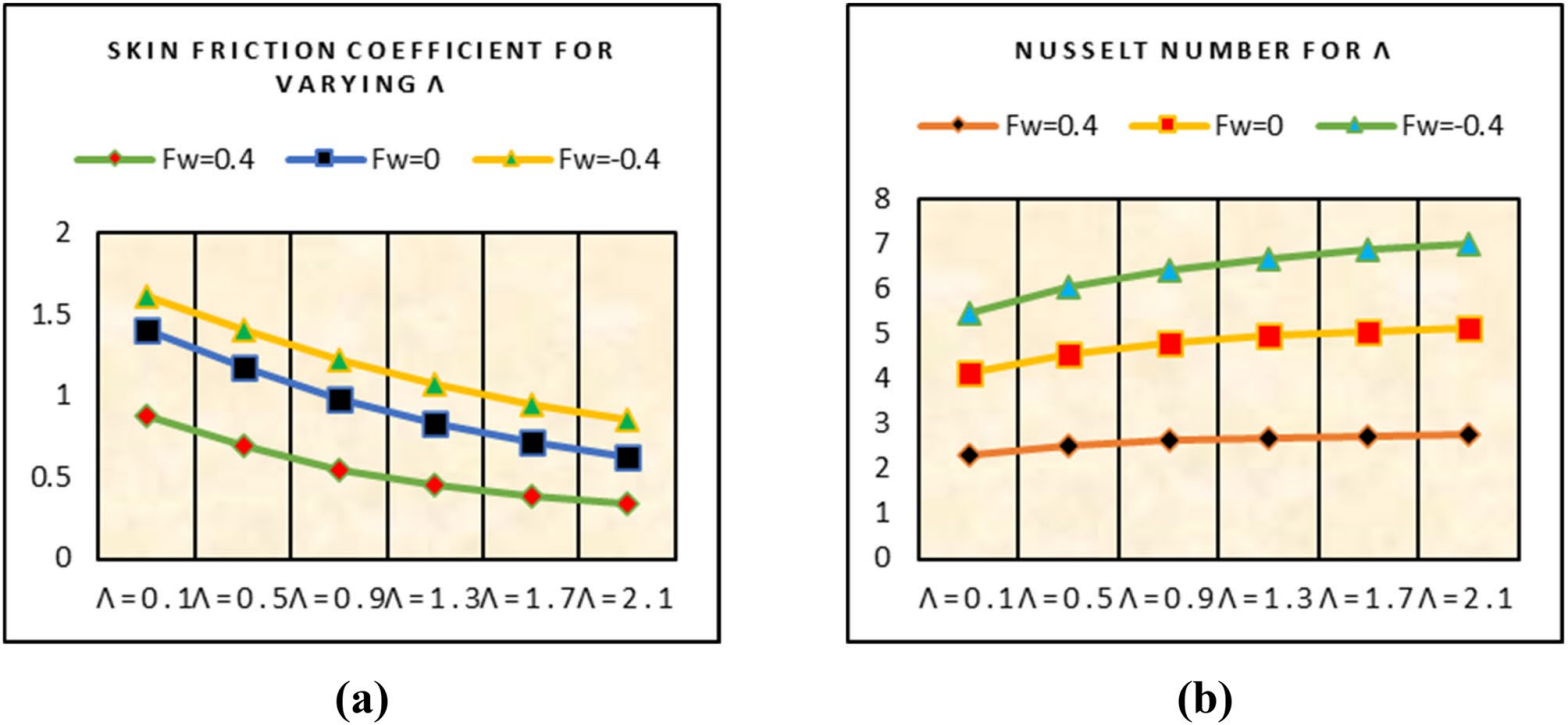
Impacts of $\lambda$ on (**a**) shear stresses (**b**) nusselt number.

**Figure 12:**
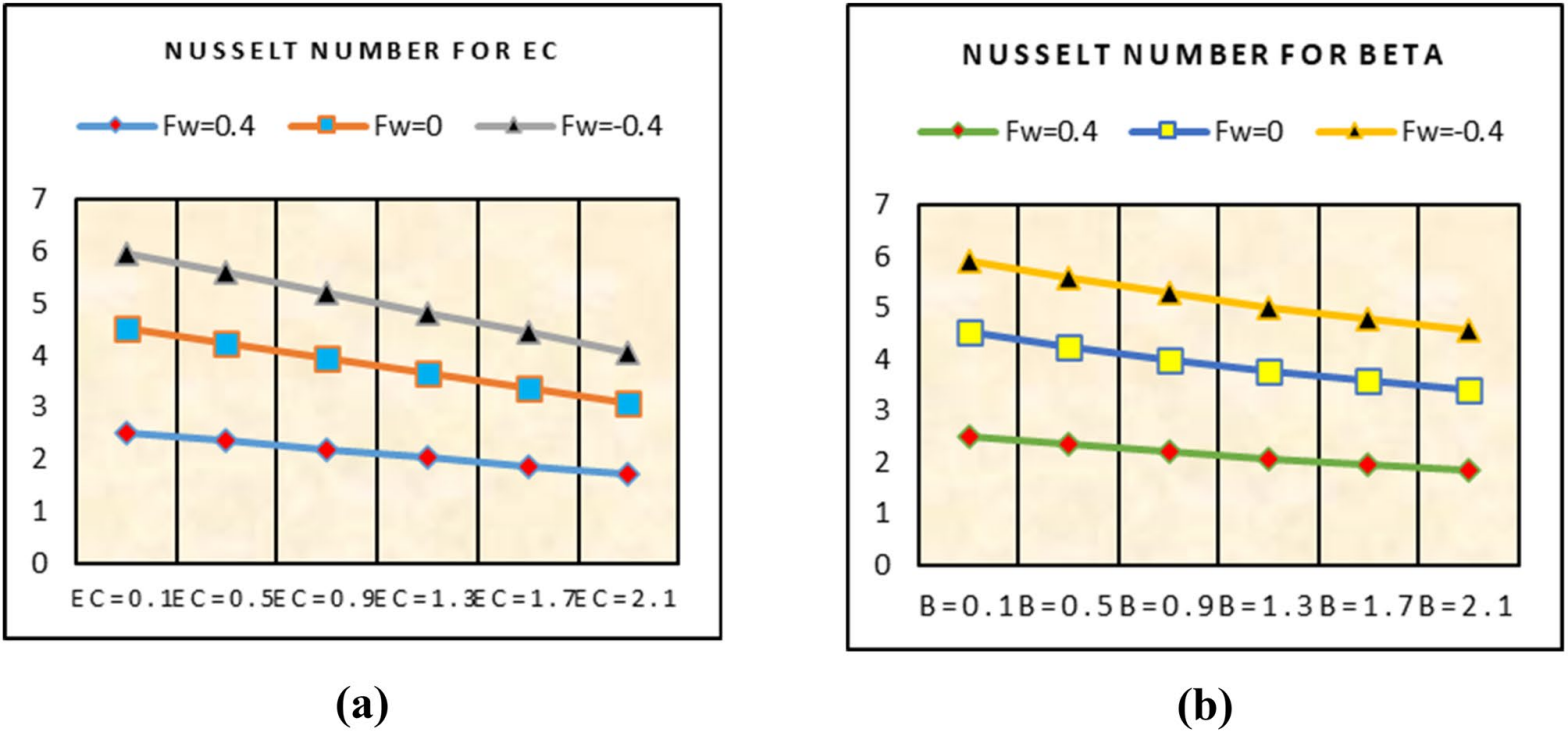
Impacts of (**a**) Ec (**b**) $\beta^{*}$ on nusselt number.

This subsection captured the trends in shear stresses and nusselt number for multiple under consideration flow parameters. From the analysis, more shear stresses on the surface are examined for suction of the fluid and stronger velocity slip effects. For injecting of the fluid, these trends become slow. Similarly, for higher velocity slip parameter more heat transfer trends at the surface are detected for suction case. These trends are painted in Fig. 11. The local heat transfer rate against more dissipative fluid and stronger thermal slip effects are plotted in Fig. 12. It is detected that the heat transfer rate at the surface declines for stronger dissipation and thermal slip effects.

### Validation of the study

This subsection conveys the comparative analysis of the present study with existing literature. The results for $C_{Fx} Re_{x}^{0.5}$ are computed and compared with Maleki et al.^6^ for the particular value of the parameters. These results are incorporated in Table 2. From the computation, the results shown an excellent agreement with Maleki et al.^6^ that validate our analysis. The computation is perfomed by taking upper limit as $\eta = 13$.

**Table 2:** Comparative analysis for $C_{Fx} Re_{x}^{0.5}$.

*f*_*w*_	*ϕ*	*λ*	Present	Maleki et al.^17^
0.5	0	0.5	0.449283	0.4493
− 0.5	0	0.5	0.180643	0.1806

## Conclusions

The flow of AA7072-Methanol and AA7075-Methanol over a radiative permeable plate under the influence of different physical flow conditions is presented. From the comprehensive discussion of the results, it is concluded that:

- The velocity of AA7072-Methanol and AA7075-Methanol abruptly increases for stronger velocity slip effects and momentum boundary layer thickness declines due to suction of the fluid.
- The temperature of the nanofluids enhances for stronger thermal radiations and heat energy inside the plate; while heat absorption opposes the fluid temperature.
- The temperature effects prevailed throughout the analysis for stronger heat generation in the plate.
- The shear stresses at the surface increases for suction of the fluid because more fluid particles transfer at the surface due to suction.
- The stronger thermal slip effects lead to drops in the local heat transfer rate for both AA7072-Methanol and AA7075-Methanol.

## Data Availability

The authors declared no additional data for this manuscript.
